# Toward Understanding the Alginate Catabolism in *Microbulbifer* sp. ALW1 by Proteomics Profiling

**DOI:** 10.3389/fbioe.2022.829428

**Published:** 2022-03-16

**Authors:** Zhipeng Li, Xiaoyi Huang, Yuxi Guo, Chenghao Zhang, Liang Yang, Xiping Du, Hui Ni, Xuchu Wang, Yanbing Zhu

**Affiliations:** ^1^ College of Ocean Food and Biology Engineering, Jimei University, Xiame, China; ^2^ Fujian Provincial Key Laboratory of Food Microbiology and Enzyme Engineering Technology, Xiamen, China; ^3^ Research Center of Food Biotechnology of Xiamen City, Xiamen, China; ^4^ Key Laboratory for Ecology of Tropical Islands, College of Life Sciences, Ministry of Education, Hainan Normal University, Haikou, China

**Keywords:** alginate catabolism, proteomics, *Microbulbifer* sp. ALW1, oligosaccharides, polysaccharides

## Abstract

The bacterial strain of *Microbulbifer* sp. ALW1 has demonstrated visible ability of degrading the cell wall of *Laminaria japonica*, and biochemical characterization has been performed on some individual enzymes to elucidate its genetic basis. However, it still remains elusive how strain ALW1 successfully breaks down the major cell wall component alginate polysaccharide and colonizes on its marine host. In this study, a mass spectrometry-based quantitative analysis of the extracellular and intracellular proteomes was introduced to elucidate the alginate degradation pathway in ALW1 strain. Mass spectrometry and biochemical assays indicated that strain ALW1 could effectively degrade alginate polysaccharide into disaccharides and trisaccharides within 12 h. Proteome analysis identified 156 and 1,047 proteins exclusively localized in extracellular and intracellular compartments, respectively, with 1,086 protein identities of dual localization. Functional annotation of the identified proteins suggested the involvement of diverse catalytic enzymes and non-catalytic molecules for the cleavage and metabolism of alginate polysaccharide. A simplified pathway was constructed to demonstrate the extracellular digestion, active transport, and intracellular conversion of alginate polysaccharide and its fragmented oligosaccharides, casting a picture of genetic loci controlling alginate catabolism by ALW1 strain. This study aims to provide a guide for utilization and genetic manipulation of the bacterial strain ALW1 for efficient alginate oligosaccharides production by fermentation.

## Introduction

Seaweeds and their constituent compounds are utilized as food, fertilizer, industrial products, medical products, and cosmetics and for producing biofuel (Wargacki et al., 2012; Kawai and Murata, 2016; Jesumani et al., 2019; Ścieszka and Klewicka, 2019; Lopez-Santamarina et al., 2020). Alginates as natural polysaccharides are major structural components of the cell wall of seaweed (Li et al., 2021). The potential applications of alginate are restricted because of its large molecular weight, low water solubility, and high solution viscosity when significant concentrations are needed. In contrast, alginate oligosaccharides (AOS), oligomers containing 2 to 25 monomers, have better bioavailability because of their improved water solubility and low solution viscosity. AOS have shown immunomodulatory, antimicrobial, antioxidant, prebiotic, antihypertensive, antidiabetic, antitumor, anticoagulant, and other activities (Liu et al., 2019; Jiang et al., 2021). With the development of marine resources, AOS have potential applications in food, cosmetics, medicine, and other fields (Roohinejad et al., 2017).

Seaweed polysaccharides can be processed into oligosaccharides through different approaches (Liu et al., 2019), including physical and chemical methods, enzymatic method (Xu et al., 2018), and fermentation (Li et al., 2017). The chemical and physical methods normally have relatively low yield of oligosaccharides, violent reactions, and certain pollutions. The enzymatic method is difficult to manipulate in industry as a result of the poor thermal tolerance and sensitivity to mechanical disturbance. Alginate depolymerization with specific microorganisms serves as an effective and indispensable method for AOS production. Alginate-degrading bacteria have been isolated and utilized for AOS production such as *Flavobacterium* sp. LXA (An et al., 2008), *Gracilibacillus* A7 (Tang et al., 2009), *Pseudoalteromonas agarovorans* CHO-12 (Choi et al., 2009), *Bacillus subtilis* KCTC 11782BP (Bang et al., 2015), and *Bacillus litoralis* M3 (Wang et al., 2016).

Proteomics is emerging as a potent tool to dissect the complex biological processes, providing direct reflection of the gene expression profiles of the organism under defined environmental conditions. *Paradendryphiella salina* has been reported to be a microorganism capable of parasitizing and degrading algal cell walls. The enzymes of polysaccharide lyase family 7 (PL7) and 8 (PL8) were identified by proteomic analysis in *P. salina*, functioning as the forefront weapon to invade and degrade alginate in cell wall to facilitate its colonization on marine host (Pilgaard et al., 2019). The two-dimensional gel electrophoresis assay uncovered the proteomic dynamics of rumen bacteria *Butyrivibrio proteoclasticus* in response to polysaccharide carbon supplies, highlighting the implication of polysaccharidases, carbohydrate-binding proteins (CBPs), ATP-binding cassette (ABC) transporter, and substrate-binding proteins (SBPs) in polysaccharide degradation and oligosaccharide uptake (Dunne et al., 2012). Proteomics has been applied to investigate the mechanism of polysaccharide degradation by microbes (Sichert et al., 2020; de Figueiredo et al., 2021; Jensen et al., 2021).

A marine strain designated *Microbulbifer* sp. ALW1 has been isolated from rotten *Laminaria japonica*, and some alginate lyases have been characterized in our previous studies (Zhu et al., 2016; Jiang et al., 2019). To have a comprehensive understanding of the biological processes of strain ALW1 breaking down alginate, the quantitative proteomics using isobaric tags for relative and absolute quantitation of the label-free strategy was employed to identify the key proteins in polysaccharide metabolism extracellularly and intracellularly. Through revealing the metabolism process of the alginate by *Microbulbifer* sp. ALW1, the informative clues obtained could facilitate the use of brown algae or its waste to produce functional materials such as AOS using microbial method, and a genetic source of polysaccharide-degrading enzymes.

## Materials and Methods

### Cell Culture

*Microbulbifer* sp. ALW1 from our laboratory stock was refreshed in 50 ml of medium A [0.5% (w/v) kelp, 3.0% (w/v) NaCl, 0.2% (w/v) K_2_HPO_4_, 0.1% (w/v) MgSO_4_•7H_2_O, 0.001% (w/v) FeSO_4_•7H_2_O] with agitation (180 rpm) at 25°C for 24 h. After the strain revival, 1 ml of the suspension was subcultured into 50 ml of medium B [0.5% (w/v) (NH_4_)_2_SO_4_, 3.0% (w/v) NaCl, 0.2% (w/v) K_2_HPO_4_, 0.1% (w/v) MgSO_4_•7H_2_O, 0.001% (w/v) FeSO_4_•7H_2_O, pH 7.5] plus 0.5% (w/v) peptone with agitation (180 rpm) at 25°C for 12 h, with the OD_600_ value of about 1.2. Subsequently, the suspension was further subcultured with 50× dilution in 1,000 ml of medium B with the addition of 0.2% (w/v) sodium alginate instead of peptone at 25°C. The cell growth was monitored by measuring the OD_600_ value. The cell metabolite reducing sugar was determined by 3, 5-dinitrosalicylic acid (DNS) (Miller, 1959). The amount of reducing sugar produced was detected by measuring the absorbance value at 540 nm. Phenol-sulfuric acid assay for total carbohydrate determination to quantify total sugars in cellular metabolites (Montgomery, 1961). The identified protein was expressed in Escherichia *coli* BL21 (DE3) according to our previous study (Zhu et al., 2020).

### HPLC-MS Analysis

Preparation of the hydrolysates from alginate lyase-treated sodium alginate was carried out according to the method described previously (Jiang et al., 2019). The samples of alginate metabolites and enzymatic hydrolysates were determined using an ultrahigh-performance liquid chromatography (Ultimate 3000, ThermoFisher Scientific, Rockford, United States) with hypersil Gold C18 column (ThermoFisher Scientific, Rockford, United States) and matrix-assisted laser desorption synaptquan ionization-time of flight mass spectrometer MS/MS (TSQ TQU04549, Thermo Fisher Scientific, Rockford, United States). The samples were determined from 150 to 800 m*/z* by mass spectrometry (MS) under positive ion mode.

### Total Protein Extraction

The cells grown for 30 h in medium B containing alginate were pelleted by centrifugation at 12000 rpm for 10 min at 4°C. The supernatant was collected and applied on a Millipore centrifugal filter 3K device (3,000 nominal molecular weight limit, Millipore, United States). The concentrated supernatant was obtained and used as the extracellular sample. The cell pellet was milled into fine powder in liquid nitrogen to obtain the intracellular sample. Then the extracellular and intracellular proteins were extracted following the modified phenol protocol as described, and air-dried pellets are dissolved in lysis buffer (7 M urea, 2 M thiourea, 2% CHAPS, 13 mM DTT) (Wang et al., 2012). Protein concentration was determined by the Bradford assay with bovine serum albumin as the standard (Bradford, 1976). The total protein extracts were stored at −20°C after lyophilization for later analysis.

### HPLC-ESI-MS/MS for Proteomics

Extracellular and intracellular proteins (300 μg each) were used for proteomic analysis, respectively. The lyophilized proteins were treated with dithiothreitol and iodoacetamide for reduction and alkylation, respectively, and then digested by trypsin following the standard procedures. Mass spectra of the peptides were acquired on an AB 5800 MALDI-TOF/TOF mass spectrometry (MS) instrument (AB SCIEX, Foster City, United States) as described (Yi et al., 2014).

### Protein Identification

Protein identification was performed with Protein Pilot™ v4.5 software (AB SCIEX, United States) against the Malus × domestica database (http://www.rosaceae.org) using the Paragon Algorithm and searching against the genome database of *Microbulbifer* sp. ALW1 (Shilov et al., 2007). Up to two missed cleavages were permitted for fully tryptic peptides. Carboxyamidomethyl cysteine and oxidized methionine were set as fixed and variable modifications, respectively. The false discovery rate (FDR) was determined by using a target-decoy search strategy (Elias and Gygi, 2007). Mass tolerance for peptide and fragment ion was 40 ppm. The acceptance of peptide assignment and protein identification was the unused ProtScore greater than 1.3 with at least two matched peptides. FDR assessment was 0.01. Three biological replicates of proteins from extracellular and intracellular samples of strain ALW1 cells cultured with alginate as the carbon source were normalized to the intensity of many qualitatively matched proteins (or peptides). All protein hits were identified with a confidence of 95%. A Venn diagram was used to identify the proteins present in all three biological replicates.

### Bioinformatics Analyses

Functional annotations of the proteins identified through label-free quantitation analysis were performed. Clusters of orthologous groups (COG) analysis of the proteins was performed for functional classification via searches in a database (http://eggnogdb.embl.de/). Gene ontology (GO) analysis was performed using the Blast2GO program (https://www.blast2go.com/) and further performed online using WEGO software (http://wego.genomics.org.cn). Kyoto Encyclopedia of Genes and Genomes (KEGG) pathway analysis was performed using the OmicShare tools, a free online platform for data analysis (http://www.omicshare.com/tools). Searches for the polysaccharide related metabolic enzymes, including glycoside hydrolases (GH), glycosyltransferases (GT), carbohydrate esterases (CE), polysaccharide lyases (PL), and auxiliary activities (AA) were performed manually on the basis of the BLASTP and the CAZy database (Lombard et al., 2014).

Following the annotation and annotation augmentation steps, the targeted proteins were blasted against KEGG GENES to retrieve the KEGG Orthology (KO) and subsequently mapped to pathways in KEGG. These metabolic pathways of alginate polysaccharide were deduced on the basis of such enzymes and the KEGG database.

### Statistical Analysis

All experiments were conducted with three technical replicates and three biological replicates. The statistical significance (*p* values) in mean values was determined with unpaired two-tailed Student’s t-test (SPSS 13.0), with *p* < 0.05 as statistically significant.

## Results and Discussion

### Growth of Strain ALW1

**FIGURE 1:**
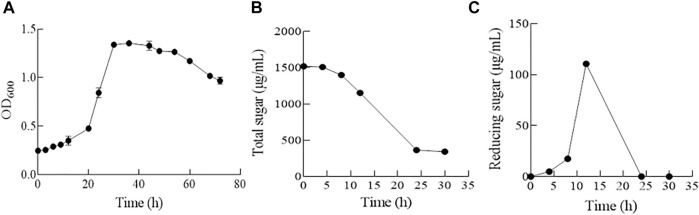
Biological growth of strain ALW1 cultured under alginate. **(A)** OD_600_, **(B)** total sugar, and **(C)** reducing sugar.

The growth dynamics of strain ALW1 cells in the medium containing alginate as the sole carbon source was recorded during the time course. The biomass was relatively stable in the first 16 h, and the OD_600_ reached the plateau of 1.36 at 30 h (Figure 1A). The total sugar content showed a downward trend and reached the lowest at about 30 h (Figure 1B). The released reducing sugar accumulated to the highest at 12 h and declined to the lowest afterward at 24 h (Figure 1C). These observations indicated that strain ALW1 could effectively break down the alginate polysaccharide to oligosaccharides and then take advantage of the degradation products. In order to coalesce the stationary phase, the cell culture at the time point of 30 h was selected for proteomic analysis.

### Analyses of Alginate Metabolites

**FIGURE 2:**
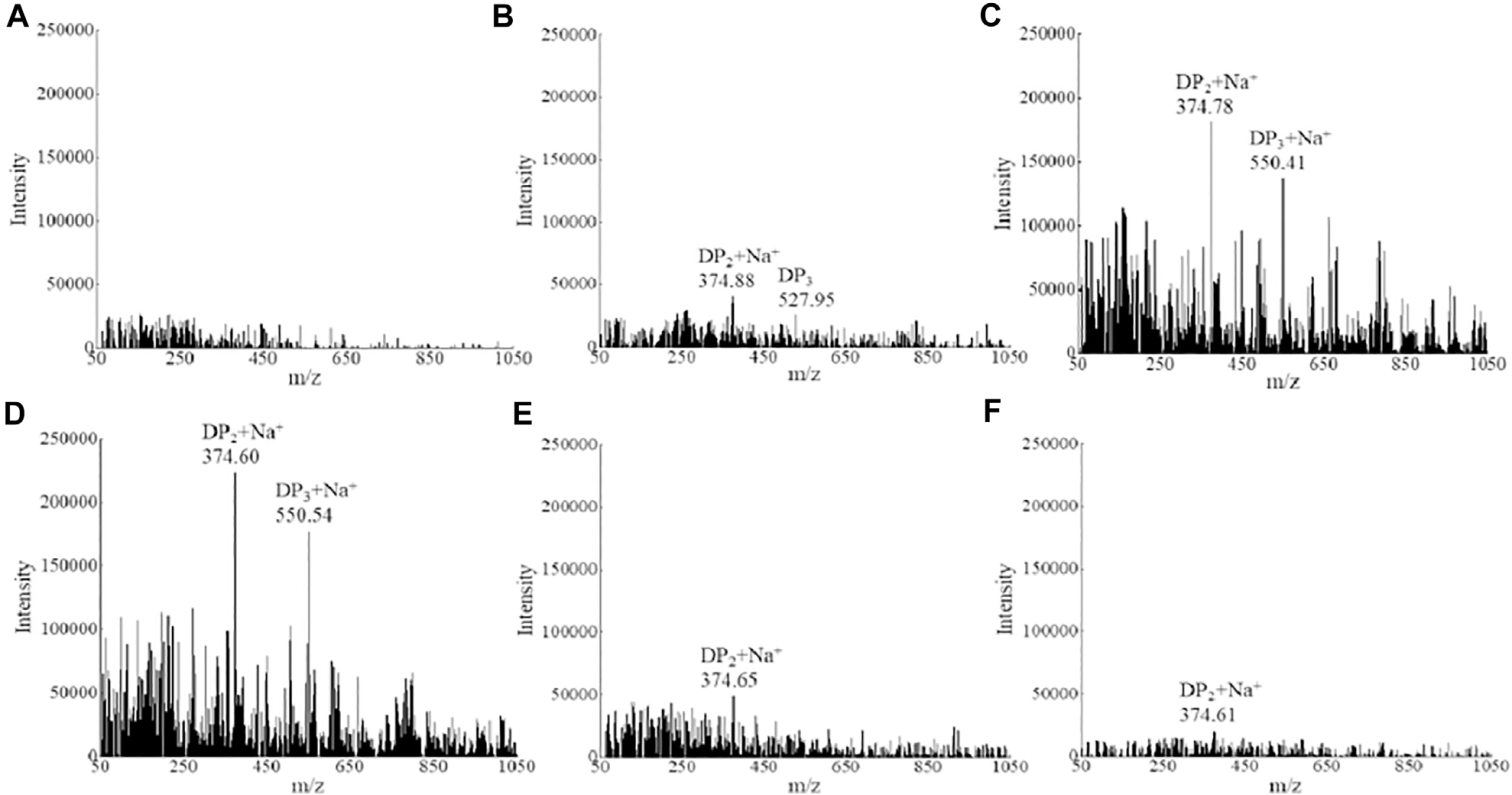
Mass spectrometry analysis of alginate metabolism. **(A)** 0 h, **(B)** 4 h, **(C)** 8 h, **(D)** 12 h, **(E)** 24 h, and **(F)** 30 h.

To have a better knowledge of the alginate degradation patterns of strain ALW1, mass spectrometry was performed to analyze the alginate metabolites over the time course. Under the positive ion mode, the degrees of polymerization (DPs) with mass-to-charge ratios (*m/z*) of the products are presumed to be two at 374 m*/z* and three at 550 m*/z*. The initial alginate polysaccharide was not detectable in this assay due to its high molecular weight (Figure 2A). After 4 h of incubation, the metabolites of disaccharides and trisaccharides showed some accumulation (Figure 2B). According to their signal intensity, the contents of disaccharides and trisaccharides reached the maximum at 12 h with the passage of time (Figure 2B−D), and showed a significant decrease at the time points of 24 and 30 h (Figures 2E,F). The dynamics presented by mass spectra was in harmony with the observation of total reducing sugar quantification, suggesting the oligosaccharides were absorbed and further metabolized by strain ALW1 cells after liberation from alginate polysaccharide. The digestion patterns are consistent with those of the previous reports (Takeshita and Oda, 2016; Tang et al., 2020). Taken together, the aforementioned observations indicated that strain ALW1 had the ability to degrade the alginate polysaccharide to produce disaccharides and trisaccharides within 12 h.

### Identification of Intracellular and Extracellular Proteins

Based on the mass spectrometry and database alignment results, the proteins were initially screened for each sample according to the rules of peptides greater than or equal to two and Unused ProtScore >1.3 (*p* value <0.05, and confidence >95%). The Venn diagram was constructed to show the distribution of the identified proteins. Specifically, 2,133 and 1,242 protein identities were shared among three biological replicates as intracellular and extracellular proteins, respectively (Supplementary Figure S1). Moreover, only 156 and 1,047 proteins were identified as exclusively extracellular and intracellular proteins within their respective groups (Supplementary Figure S2), indicating that most of extracellular proteins were secreted from intracellular synthesis. It is worth mentioning that the intracellular involvement of alginate metabolism was significantly more than that of the extracellular counterpart, as the intercellular proteins far outnumbered the extracellular proteins. It is presumable that the intracellular environment provides a more favorable niche for enzyme activities, implying the more vigorous intracellular metabolic activities of strain ALW1.

### Protein Functional Classification

**FIGURE 3:**
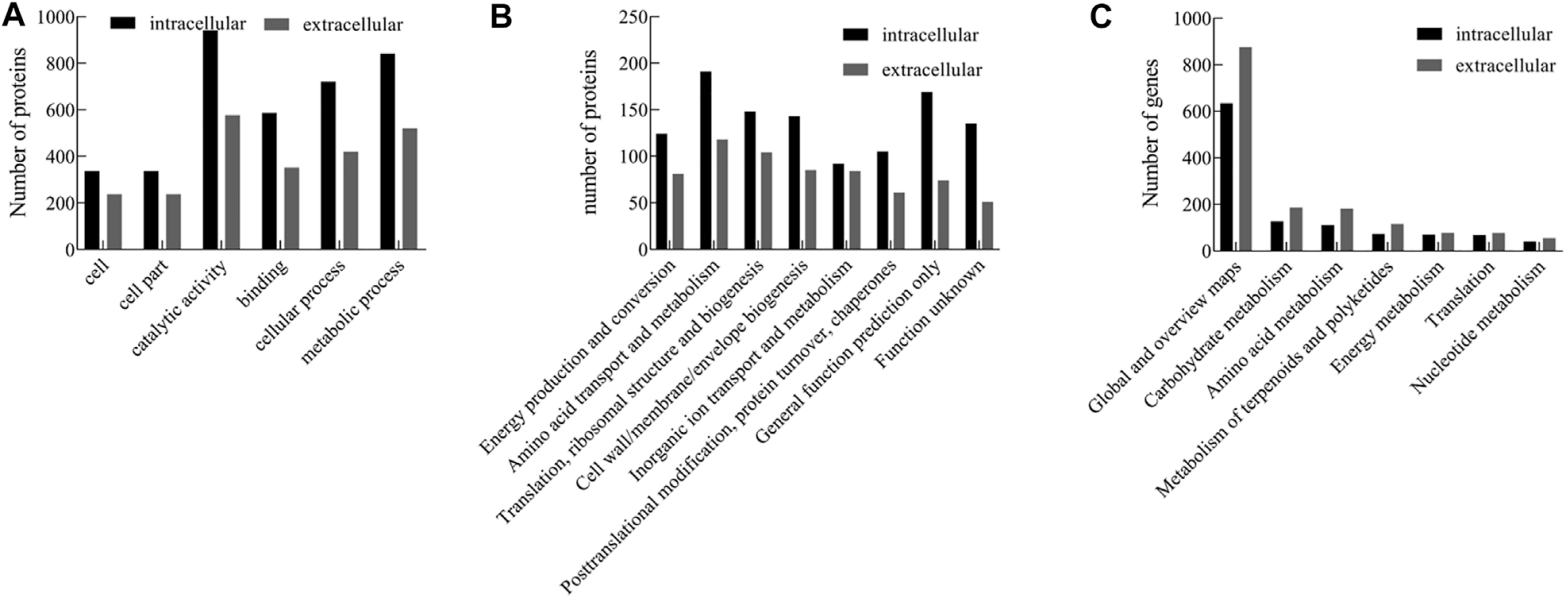
GO **(A)**, COG **(B)**, and KEGG **(C)** pathway analysis of intracellular and extracellular proteins from *Microbulbifer* sp. ALW1.

To identify the significantly enriched GO functional groups of the intracellular and extracellular proteins of strain ALW1 cells, WEGO software was used to perform a GO analysis and confirm their classifications. According to the GO annotations, the subcategories of the proteins were listed under different terms, including biological process, cellular component, and molecular function. The results of GO analysis showed that strain ALW1 metabolized alginate polysaccharide mainly concentrated in catalytic activity (GO:0003824), metabolic process (GO:0008152), cellular process (GO: 0009987), binding (GO: 0005488), cell part (GO: 0044464), and cell (GO: 0005623) (Figure 3A). Notably, the proteins under catalytic activity and metabolic processes were predominant, especially for the intracellular proteins, substantiating the biological relevance of strain ALW1 in alginate decomposition. In addition, a significant portion of proteins was related to binding for the intracellular and extracellular proteins. As polysaccharides may serve as the main carbon source for many marine bacteria (Alderkamp et al., 2007; Kabisch et al., 2014; Sichert et al., 2020), proteins encoded in operons known as polysaccharide utilization loci (PULs) are often enriched to counter the physical barrier of a wide variety of polysaccharides, functioning as carbohydrate-degrading enzymes and binding proteins (Adams et al., 2021). The proteins related to cellular process (GO: 0009987) may participate in material transportation, information transmission, and the maintenance of basic life activities.

The intracellular and extracellular proteins of *Microbulbifer* sp. ALW1 were further grouped according to their main biological activities, as defined by the functional catalog of COG. The functional categories of intracellular proteins mainly included amino acid transport and metabolism, general function prediction only, translation, ribosomal structure and biogenesis, cell wall/membrane/envelope biogenesis, and function unknown (Figure 3B). Meanwhile, the extracellular counterparts were mainly amino acid transport and metabolism, translation, ribosomal structure, biogenesis, cell wall/membrane/envelope biogenesis, and inorganic ion transport and metabolism. In addition, there was a minority of intracellular and extracellular proteins under the category of posttranslational modification, protein turnover, and chaperones, which normally played essential roles in regulating protein fate and modulating protein functions (Latko et al., 2019).

To further investigate the biological functions of these proteins, KEGG pathway analysis was performed on the identified proteins that were mapped to the reference canonical pathways in the KEGG database. The results revealed that the extracellular and intracellular proteins of strain ALW1 were mainly mapped in global and overview maps, with low protein enrichment in the pathways such as carbohydrate metabolism, amino acid metabolism, metabolism of terpenoids and polyketides, energy metabolism, translation, and nucleotide metabolism (Figure 3C). The protein enrichment patterns indicated that the majority of the proteins were presenting the overall picture of cell metabolism.

### Identification of CAZymes

**FIGURE 4:**
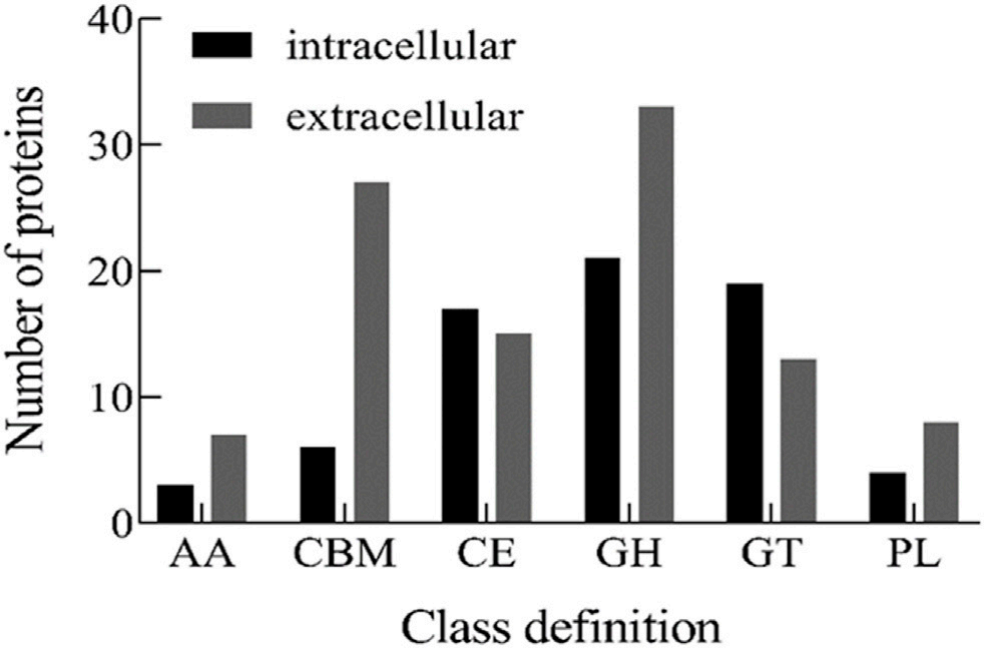
CAZy classification of intracellular and extracellular proteins from *Microbulbifer* sp. ALW1 cultured with alginate. AA means auxiliary activity. CBM means carbohydrate-binding module. CE means carbohydrate esterase. GH means glycoside hydrolases. GT means glycosyltransferases. PL means polysaccharide lyase.

CAZymes represent the carbohydrate-active enzymes to digest diverse sugar bonds in glycans (Kunath et al., 2017). To determine the key enzymes involved in alginate degradation by strain ALW1, the CAZymes analysis was performed for the intracellular and extracellular proteins of this strain. The distribution map of CAZymes indicated that except for glycosyltransferases (GT) and carbohydrate esterases (CE), the numbers of the other types including the non-catalytic protein carbohydrate-binding modules (CBMs) of extracellular proteins were greater than those of intracellular proteins (Figure 4), indicating the robust extracellular enzymatic activities of breaking down the alginate polysaccharide. In addition, among these carbohydrate-active enzymes, the GH were predominant over the other major types of CAZymes, and the PL were the least abundant (Figure 4). GH process the common carbohydrate of motif alpha-linked glycoside in nature (McGuire et al., 2020). PL are mainly responsible for the breakdown of polysaccharides into oligosaccharides and monomers through exolytic and endolytic cleavages (Li et al., 2021), and the resulting oligosaccharides can be transported into the bacterial cells via the transport systems. CBM is carbohydrate-active protein with a discrete fold having carbohydrate-binding activity (Shoseyov et al., 2006). There was a greater number of GH, PL, and CBM in the extracellular secretion than in the intracellular compartments (Figure 4), reflecting the active digestion of alginate polysaccharide extracellularly before the further actions intracellularly upon the uptake of their metabolites.

### Alginate Polysaccharide Metabolism Pathway in Strain ALW1

Based on KEGG pathway analysis, some proteins with key signatures to play roles in alginate polysaccharide metabolism were summarized to decipher the potential mechanism of alginate degradation and adsorption in strain ALW1 (Table 1). The secreted enzymes included alginate lyase (protein ID: 1_423, 1_424 and 1_430), arabinan endo-1,5-alpha-l-arabinosidase (protein ID: 1_1147), alpha-glucosidase (protein ID: 1_3641), oligosaccharide 4-alpha-d-glucosyltransferase (protein ID: 1_3668), and beta-glucosidase (protein ID: 1_2974), which are reported to function in degrading seaweed polysaccharides into oligosaccharide fragments or monosaccharide molecules (Zehavi et al., 1992; Wong et al., 2000; Zhu et al., 2019; Tang et al., 2020; Li et al., 2021). In addition, some transporters were identified, including ABC transporter (protein ID: 1_960 and 1_1141), major facilitator superfamily (MFS) transporter (protein ID: 1_426 and 1_2094), and TonB system transport protein (protein ID: 1_1049 and 1_1051), which may function as the vehicles (Matthews et al., 2000; Hollenstein et al., 2007; Yan, 2013; Maruyama et al., 2015; Reintjes et al., 2017) for the uptake of primary metabolites of alginate polysaccharide by strain ALW. After adsorption by strain ALW1 cells with the assistance of the transporters, the oligosaccharides and monomers were further degraded through different intracellular alginate lyases and glycolysis pathway.

**TABLE 1:** Proteins about the polysaccharide metabolism by strain ALW1.

Pathway	K_ID	Gene ID	Description	Intracellular	Extracellular
Glycolysis/gluconeogenesis	K00121	1_1231	Alcohol dehydrogenase	+	+
	K00128	1_1766	ALDH; aldehyde dehydrogenase (NAD^+^)	+	+
	K00134	1_2660	GAPDH, gapA; glyceraldehyde 3-phosphate dehydrogenase	+	+
	K00134	1_3294	GAPDH, gapA; glyceraldehyde 3-phosphate dehydrogenase	+	+
	K00138	1_1002	aldB; aldehyde dehydrogenase	+	
	K00161	1_532	PDHA, pdhA; pyruvate dehydrogenase E1 component alpha subunit	+	+
	K00162	1_533	PDHB, pdhB; pyruvate dehydrogenase E1 component beta subunit	+	
	K00163	1_1464	aceE; pyruvate dehydrogenase E1 component	+	+
	K00382	1_1927	DLD, lpd, pdhD; dihydrolipoamide dehydrogenase	+	+
	K00627	1_1465	DLAT, aceF, pdhC; pyruvate dehydrogenase E2 component dihydrolipoamide acetyltransferase	+	+
	K00627	1_534	DLAT, aceF, pdhC; pyruvate dehydrogenase E2 component dihydrolipoamide acetyltransferase	+	
	K00845	1_3690	glk; glucokinase	+	+
	K00850	1_1539	pfkA, PFK; 6-phosphofructokinase 1	+	+
	K00873	1_3293	PK, pyk; pyruvate kinase	+	
	K00873	1_1390	PK, pyk; pyruvate kinase	+	+
	K00927	1_921	PGK, pgk; phosphoglycerate kinase	+	+
	K01610	1_708	E4.1.1.49, pckA; phosphoenolpyruvate carboxykinase (ATP)	+	+
	K01624	1_922	FBA, fbaA; fructose-bisphosphate aldolase, class II	+	+
	K01689	1_2964	ENO, eno; enolase	+	+
	K01792	1_1487	E5.1.3.15; glucose-6-phosphate 1-epimerase	+	
	K01803	1_1361	TPI, tpiA; triosephosphate isomerase (TIM)	+	+
	K01810	1_3164	GPI, pgi; glucose-6-phosphate isomerase	+	+
	K01895	1_830	ACSS, acs; acetyl-CoA synthetase	+	+
	K01895	1_3233	ACSS, acs; acetyl-CoA synthetase	+	
	K15633	1_773	gpmI; 2,3-bisphosphoglycerate-independent phosphoglycerate mutase	+	+
	K15778	1_613	pmm-pgm; phosphomannomutase/phosphoglucomutase	+	+
Polysaccharide metabolism	K00850	1_1539	pfkA, PFK; 6-phosphofructokinase 1	+	+
	K00971	1_3339	manC, cpsB; mannose-1-phosphate guanylyltransferase	+	
	K00971	1_3515	manC, cpsB; mannose-1-phosphate guanylyltransferase	+	
	K01624	1_922	FBA, fbaA; fructose-bisphosphate aldolase, class II	+	+
	K01729	1_423	algL; poly (beta-d-mannuronate) lyase	+	+
	K01729	1_430	Alginate lyase; F5/8 type C domain poly (beta-d-mannuronate) lyase	+	
	—	1_424	Alginate lyase [*Microbulbifer agarilyticus*] poly (beta-d-mannuronate)		+
	—	1_425	KdgF, pectin degradation protein [*Dickeyadadantii*]	+	+
	K00874	1_428	KdgK, ketodeoxygluconockinase [*M. agarilyticus*]	+	+
	K01187	1_3710	Glycoside hydrolase 97; oligomerization	+	+
	K01803	1_1361	TPI, tpiA; triosephosphate isomerase (TIM)	+	+
	K01808	1_3455	rpiB; ribose 5-phosphate isomerase B	+	+
	K01840	1_3508	manB; phosphomannomutase	+	+
	K07026	1_1738	E3.1.3.70; mannosyl-3-phosphoglycerate phosphatase	+	
	K06113	1_1147	Arabinan endo-1,5-alpha-l-arabinosidase [EC:3.2.1.99]		+
	K01187	1_3641	Alpha-glucosidase		+
	K18820	1_3668	Oligosaccharide 4-alpha-d-glucosyltransferase		+
	K05349	1_2974	Beta-glucosidase		+
	K01805	1_114	xylA; xylose isomerase	+	+
	K15778	1_613	pmm-pgm; phosphomannomutase/phosphoglucomutase	+	+
Transporters	K02058	1_1141	sugar ABC transporter substrate-binding protein	+	+
	K00831	1_2094	MFS transporter [*M. agarilyticus*]		+
	K08191	1_426	MFS transporter [*Shewanella frigidimarina*]		+
	K03561	1_1051	TonB system transport protein ExbB2		+
	—	1_1049	Biopolymer transporter TonB		+
	K06147	1_960	ABC transporter ATP-binding protein		+

To further verify the function of alginate lyases, the putative alginate lyase of protein ID 1_430 was expressed in *E. coli* BL21 (DE3) and applied to degrade sodium alginate. Under the positive ion mode, the mass spectra revealed that alginate lyase 1_430-mediated degradation of alginate generated a mixture of DP2 (disaccharides), DP3 (trisaccharides), and DP4 (tetrasaccharides) (Supplementary Figure S3). The molecular weight of the alginate lyase was 71.5 kDa, and the specific activity of enzyme could reach 26.98 U/mg. In addition, the other alginate lyases have been obtained from *Microbulbifer* sp. ALW1 in our previous study. These results further verified the ability of strain ALW1 to metabolize alginate polysaccharide to disaccharides and trisaccharides, while the monosaccharide was predicted to be absorbed into the cell and further utilized and was not detected extracellularly (Figure 2).

**FIGURE 5:**
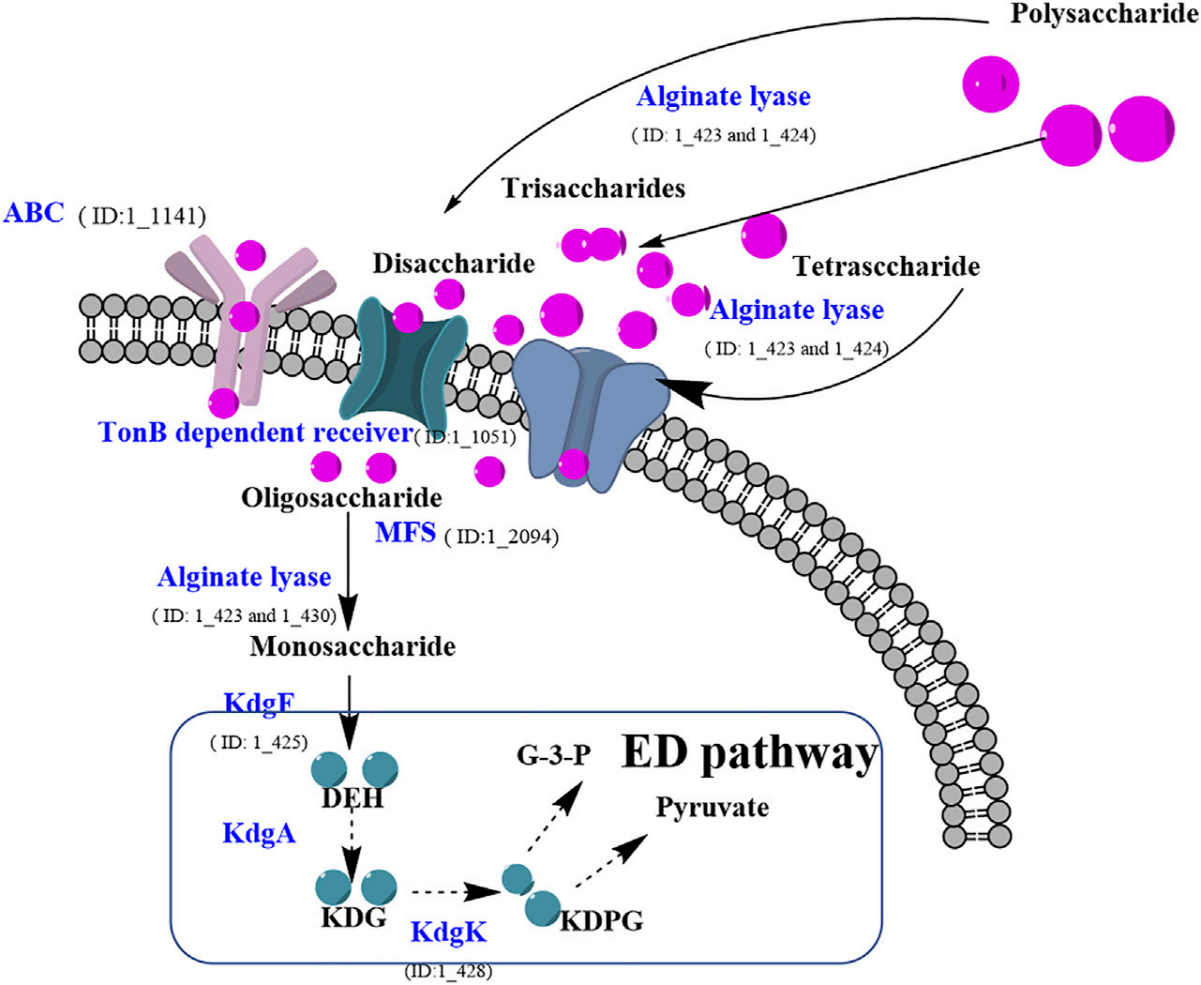
Prediction of alginate polysaccharide metabolic pathway of *Microbulbifer* sp. ALW1. MFS means major facilitator super family. DEH means 4-deoxy-l-erythro-5-hexoseulose urinate. KDPG means 2-keto-3-deoxy-6-phosphate-gluconic acid. KDG means 2-keto-3-deoxy-d-gluconic acid. KdgK means 2-keto-3-deoxygluconate kinase. G-3-P means glyceraldehyde-3-phosphate.

After integration of the molecular players and their potential nodes in the signaling pathways, the alginate polysaccharide metabolism pathway in strain ALW1 was sketched (Figure 5). Generally, upon the feeding of alginate polysaccharide, *Microbulbifer* sp. ALW1 produces some extracellularly localized enzymes collectively named alginate lyases and the related hydrolases and transferases to break down the polymers into smaller fragments and monomers. The oligosaccharide and monosaccharide are further actively transported into the periplasmic space of strain ALW1 cells. The monosaccharide is non-enzymatically converted and further reduced to 2-keto-3-deoxy-d-gluconic acid (KDG) subsequently entering into the Entner–Doudoroff (ED) pathway (Takase et al., 2014; Hobbs et al., 2016). In this pathway, KDG is utilized by a two-step catalytic reaction under the sequential actions of 2-keto-3-deoxygluconate aldolase (KdgA) and kinase (KdgK) to produce glyceraldehyde-3-phosphate and pyruvate (Hobbs et al., 2016; Zhang et al., 2021), which are metabolized through tricarboxylic acid (TCA) cycle to support cell growth.

## Conclusion

The dynamic patterns of the metabolite accumulation of alginate after digestion by *Microbulbifer* sp. ALW1 demonstrated its ability to fragment polysaccharide into oligosaccharides. The intracellular and extracellular proteome of strain ALW1 fed with an alginate carbon source presented the overall picture of the orchestration of polysaccharide breakdown, transport, and cellular metabolism. The identification of diverse enzymes and other molecular players involved in the catabolic pathway accentuates the complex actions of alginate degradation to produce oligosaccharides and to support cell growth. This study provides references for genetic engineering of the bacterial strain to enhance its performance in industrial fermentation to produce the bioactive AOS.

## Data Availability

The original contributions presented in the study are included in the article/Supplementary Material, further inquiries can be directed to the corresponding authors.
