# Supplementary figures and images for: Toward Understanding the Alginate Catabolism in Microbulbifer sp. ALW1 by Proteomics Profiling
Source: Front Bioeng Biotechnol. 2022 Mar 16;10:829428. doi: 10.3389/fbioe.2022.829428 (PMC8967155; doi:10.3389/fbioe.2022.829428)

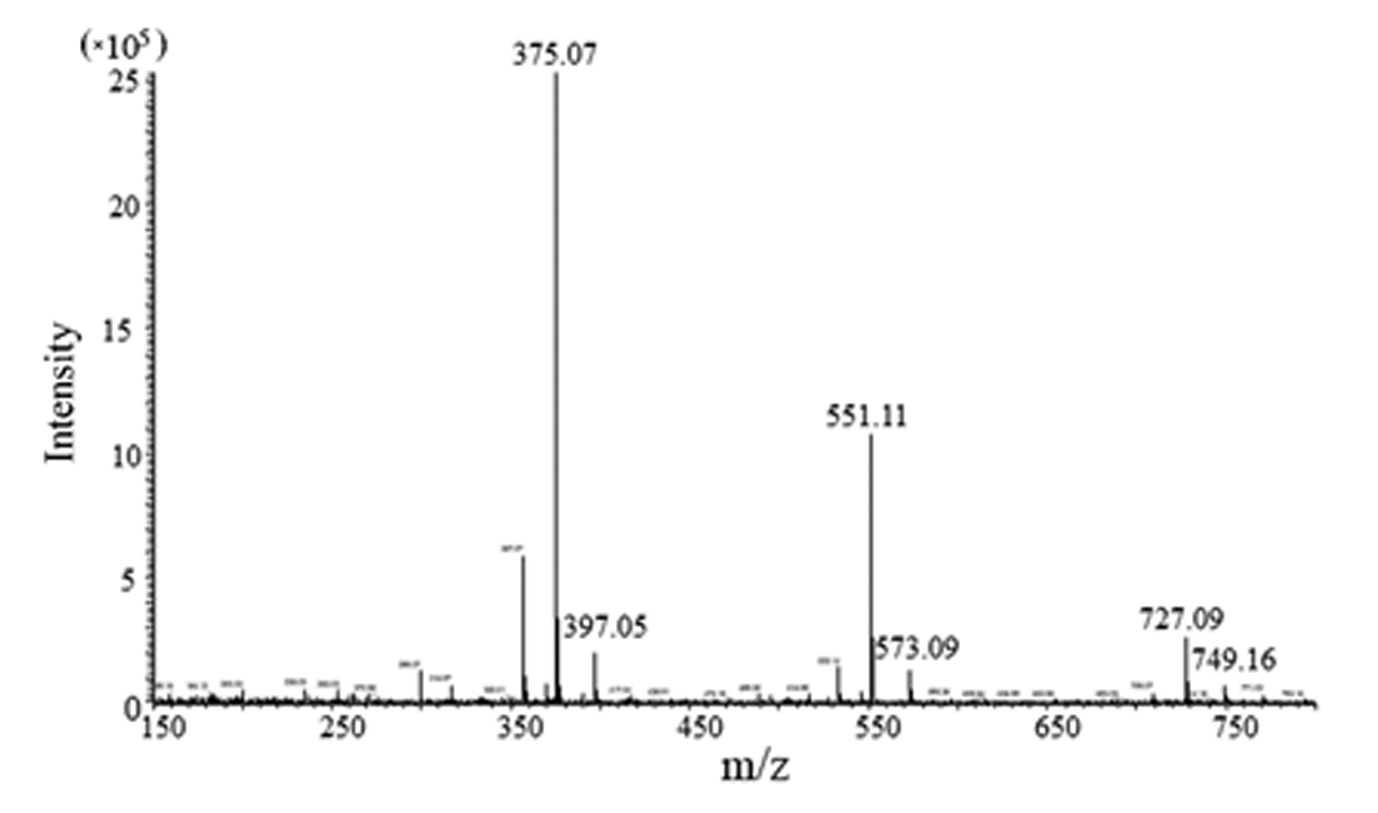

Supplement: Supplementary file 1 [file Image3.tif]

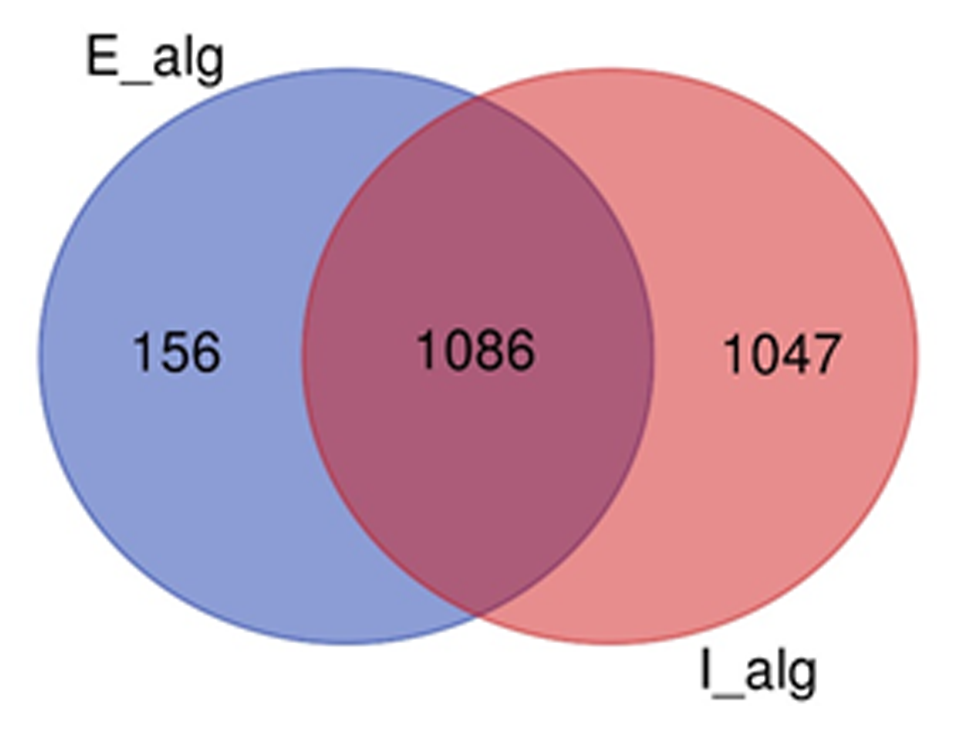

Supplement: Supplementary file 2 [file Image2.tif]

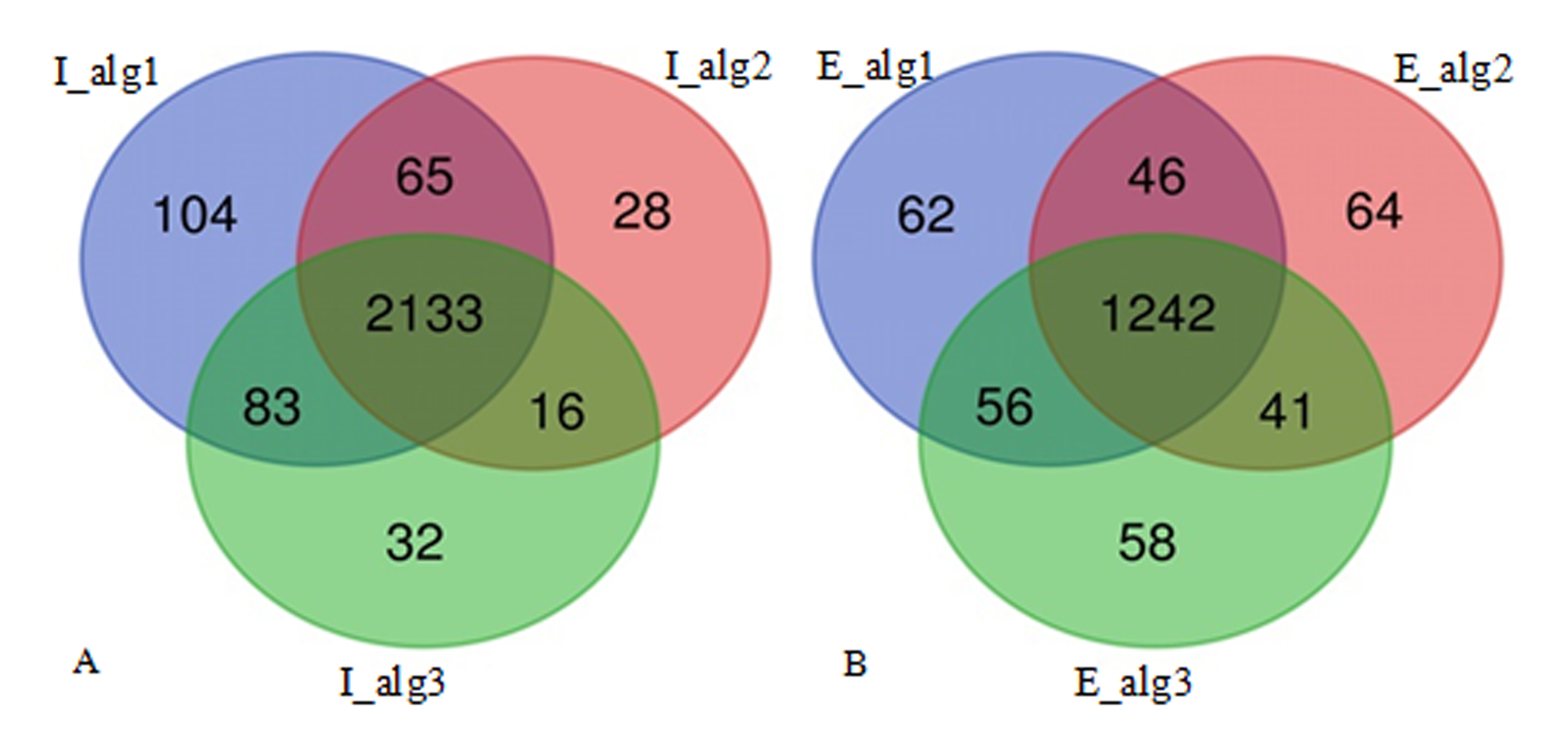

Supplement: Supplementary file 3 [file Image1.tif]
